# Automated Machine Learning to Develop Predictive Models of Metabolic Syndrome in Patients with Periodontal Disease

**DOI:** 10.3390/diagnostics13243631

**Published:** 2023-12-08

**Authors:** Ovidiu Boitor, Florin Stoica, Romeo Mihăilă, Laura Florentina Stoica, Laura Stef

**Affiliations:** 1 Dental Medicine Research Center, Faculty of Medicine, “Lucian Blaga” University, 550024 Sibiu, Romania; ovidiu.boitor@gmail.com; 2 Department of Mathematics and Informatics, Research Center in Informatics and Information Technology, Faculty of Sciences, “Lucian Blaga” University, 550024 Sibiu, Romania; laura.cacovean@ulbsibiu.ro; 3 Department of Internal Medicine, Faculty of Medicine, “Lucian Blaga” University, 550024 Sibiu, Romania; romeo.mihaila@ulbsibiu.ro; 4 Department of Oral Health, Dental Medicine Research Center, Faculty of Medicine, “Lucian Blaga” University, 550024 Sibiu, Romania; laura.stef@ulbsibiu.ro

**Keywords:** periodontal disease, metabolic syndrome, AutoML, SHAP, predictive model

## Abstract

Metabolic syndrome is experiencing a concerning and escalating rise in prevalence today. The link between metabolic syndrome and periodontal disease is a highly relevant area of research. Some studies have suggested a bidirectional relationship between metabolic syndrome and periodontal disease, where one condition may exacerbate the other. Furthermore, the existence of periodontal disease among these individuals significantly impacts overall health management. This research focuses on the relationship between periodontal disease and metabolic syndrome, while also incorporating data on general health status and overall well-being. We aimed to develop advanced machine learning models that efficiently identify key predictors of metabolic syndrome, a significant emphasis being placed on thoroughly explaining the predictions generated by the models. We studied a group of 296 patients, hospitalized in SCJU Sibiu, aged between 45–79 years, of which 57% had metabolic syndrome. The patients underwent dental consultations and subsequently responded to a dedicated questionnaire, along with a standard EuroQol 5-Dimensions 5-Levels (EQ-5D-5L) questionnaire. The following data were recorded: DMFT (Decayed, Missing due to caries, and Filled Teeth), CPI (Community Periodontal Index), periodontal pockets depth, loss of epithelial insertion, bleeding after probing, frequency of tooth brushing, regular dental control, cardiovascular risk, carotid atherosclerosis, and EQ-5D-5L score. We used Automated Machine Learning (AutoML) frameworks to build predictive models in order to determine which of these risk factors exhibits the most robust association with metabolic syndrome. To gain confidence in the results provided by the machine learning models provided by the AutoML pipelines, we used SHapley Additive exPlanations (SHAP) values for the interpretability of these models, from a global and local perspective. The obtained results confirm that the severity of periodontal disease, high cardiovascular risk, and low EQ-5D-5L score have the greatest impact in the occurrence of metabolic syndrome.

## 1. Introduction

Periodontal disease is a chronic inflammatory condition that affects the tissues surrounding the teeth. Recent studies recognize periodontal disease as a pressing public health concern, primarily due to their frequent connection with metabolic disorders. Treating these conditions entails substantial healthcare costs and can also lead to a diminished quality of life for affected individuals [1].

The initial clinical sign of periodontal disease is typically seen in the form of gingivitis, which is marked by swollen gums, bleeding when brushing teeth, and mild discomfort. Without intervention, this condition frequently advances into deeper gum tissues and inflammation of the periodontium, leading to periodontitis [2].

Numerous studies conducted over the past few decades have consistently highlighted a frequent association between systemic diseases such as diabetes, obesity, and cardiovascular conditions [3,4,5], establishing a clinical relationship between metabolic syndrome and periodontal disease [6,7].

Metabolic syndrome encompasses a cluster of risk factors associated with cardiovascular disease and type 2 diabetes, occurring concurrently at a frequency higher than expected by random chance [8].

The National Cholesterol Education Program Adult Treatment Panel III (NCEP ATP III) provided the most used definition of metabolic syndrome [9]. According to this definition, an individual must exhibit at least three of the following risk factors: (a) increased abdominal circumference, (b) reduced levels of high-density lipoprotein (HDL) cholesterol in the plasma, (c) elevated plasma triglyceride levels, (d) hypertension, and (e) increased glucose levels [10].

Inadequate oral hygiene within an area affected by gingivitis can result in the proliferation of bacteria and the development of a pathogenic subgingival biofilm primarily composed of anaerobic bacteria, including Porphyromonas gingivalis and Treponema forsythia [11,12].

Published studies have provided confirmation that the presence of periodontal disease in individuals with diabetes, obesity, and cardiovascular conditions heightens the risk of thrombosis in vital organs such as the heart, brain, lungs, or kidneys [13,14,15].

The bacteria found within the pathogenic subgingival biofilm trigger an inflammatory response, leading to the release of pro-inflammatory biomarkers. Some of these biomarkers enter the saliva, while others enter the bloodstream, resulting in a persistent, low-level inflammatory condition in the body [13,16].

Periodontitis elevates the levels of inflammatory cytokines like interleukin-6 (IL-6), interleukin-1 (IL-1) [17,18], and tumor necrosis factor alpha (TNF-α) [19]. The C-reactive protein (CRP) is synthesized by the liver in response to these inflammatory cytokines. Results from [20] confirm an elevation of CRP levels in periodontitis patients, and this is a known predictor of type 2 diabetes and cardiovascular disease risk [18,20]. Given that CRP is now considered a biomarker of systemic inflammation and that metabolic syndrome is associated with systemic inflammation [21], researchers have aimed to assess how periodontitis might impact the onset, development, or progression of metabolic syndrome.

The meta-analysis from [22], dedicated to the link between periodontal disease and the risk of metabolic syndrome, included 39 studies with crude odds ratios and 35 studies with adjusted odds ratios (calculated individually). The pooled crude and adjusted odds ratios were calculated as weighted average of the individual odds ratios from the respective studies. The results demonstrated an association between periodontitis and metabolic syndrome with a pooled crude odds ratio of 1.99 (95% confidence interval: 1.75–2.25) and a pooled adjusted odds ratio of 1.46 (95% confidence interval: 1.31–1.61).

A cross-sectional study detailed in [23] concludes that increased gingival index and high depth of periodontal pockets were associated with increased triglycerides levels and low HDL cholesterol.

As consequences of periodontal disease, hyperactivation of neutrophils and expression of proinflammatory cytokines (adipokines, IL-1-beta, TNF-α) leads to insulin resistance [24]. As a result, the body endeavors to counteract heightened insulin resistance by augmenting insulin secretion, which is proved by elevated insulin levels (hyperinsulinemia) observed in individuals with periodontitis [25]. Due to its role as an anabolic hormone, insulin facilitates glucose uptake and fat storage. Therefore, the presence of hyperinsulinemia contributes to the promotion of obesity [26], hypertension, and hyperglycemia [27,28].

Specialist dentists can quantify periodontal inflammation using various procedures, including measuring the depth of periodontal pockets, evaluating spontaneous bleeding, assessing epithelial insertion loss, and utilizing dental and periodontal hygiene indices recommended by the World Health Organization (WHO) [29].

Numerous recent studies suggest that the combination of periodontal disease with other risk factors such as obesity, smoking, arterial hypertension, type 2 diabetes, hypercholesterolemia, and a sedentary lifestyle can potentially double the risk of thrombotic cardiovascular accidents [1,15,16].

This research delves into the association between periodontal disease and metabolic syndrome, integrating information about general health status and overall well-being. Our objective was to create highly optimized machine learning models capable of identifying significant predictors of metabolic syndrome, with a notable emphasis on providing comprehensive explanations for the generated predictions.

Machine learning (ML) methods have the capability to address the limitations of traditional regression models [30] and have been successfully applied by recent studies in domains which span different medical specialties and involves a complex interplay of biological, environmental, and lifestyle factors.

In the machine learning approach, the effort is shifted from a deep understanding of the application domain to the construction, optimization, and validation of models. Techniques like bootstrap aggregating, boosting, averaging, model stacking, or cascading combine many simpler models into one complex model to obtain better predictive performance.

The advantages of complex models are obvious in the accuracy of the results obtained, but there are also disadvantages that must be addressed. Complex models can appear to function as “black boxes”. Typically, the term “black-box” model is used for models with a complex structure that is difficult for humans to understand. It can be difficult, if not impossible, to explain how thousands of variables affect a model’s prediction.

The structure of a complex model, such as, for example, deep ensemble model [31], may be opaque and, consequently, it can be difficult to decide whether the model is consistent with the application domain. An analysis of real problems with complex black-box models can be found in O’Neil [32].

As black-box Machine Learning (ML) models are more commonly utilized for making crucial predictions in critical contexts, there is a growing demand for transparency from various stakeholders in the field of AI [33]. In [34] it is mentioned that proliferation of “black-box” algorithms presents many challenges for companies and governments seeking to comply with the General Data Protection Regulation (GDPR), from the perspective of the “Right to Explanation”, i.e., the right to be provided with an explanation for an output of an automated algorithm.

Generally, people are hesitant to embrace techniques that lack direct interpretability, manageability, and reliability [33]. In order to address these problems, Explainable Artificial Intelligence (XAI) proposes a set of techniques, methods, and tools that aim to make the outputs and decisions of artificial intelligence (AI) systems understandable and interpretable by humans. XAI is necessary for addressing the challenges associated with deploying complex AI systems in real-world applications. It helps improve transparency, trust, and accountability while also enabling better collaboration between humans and AI systems.

Explanations that underpin a model’s output are vital, particularly in fields like medical diagnoses. In such contexts, experts need more comprehensive information from the model beyond a basic binary prediction to assist in validating and supporting their diagnosis. If experts can understand how a model arrived at a particular decision, they are more likely to trust the system.

In this research, we used the SHapley Additive exPlanations (SHAP) framework [35] to provide explanations for the output of machine learning models. A differentiation can be made between interpretability and explainability, but same as in [36] we will refrain from doing this in order to prevent unnecessary use of technical language for medical experts. SHAP values offer a way to interpret and understand the output of complex machine learning models, making them more transparent and trustworthy. The framework helps identify the importance of each feature in the model’s decision-making process. This is crucial for understanding which factors have the most influence on the predictions. In applications like healthcare or medical diagnosis, where model predictions have significant consequences, the SHAP framework can offer valuable insights to support decision-making by providing a clear rationale for each prediction. The SHAP framework plays a crucial role in making machine learning models more interpretable and facilitating the responsible and ethical use of AI systems [37].

We proposed the use of the EuroQol 5-Dimensions 5-Levels (EQ-5D-5L) score as the feature variable. The EQ-5D-5L is a widely used generic preference-based Health-Related Quality of Life (HRQoL) questionnaire [38]. The EQ-5D-5L is an updated version of the EQ-5D-3L questionnaire, with the aim of enhancing its sensitivity and mitigating ceiling effects in comparison to the EQ-5D-3L. Both are tools designed to assess an individual’s health status and overall well-being. More precisely, the state of health of a person is evaluated in five dimensions: mobility, self-care, usual activities, pain/discomfort, and anxiety/depression. In the case of the EQ-5D-3L questionnaire, for each dimension, respondents choose from three levels of response: no problems, some problems, and extreme problems. The key difference between EQ-5D-3L and EQ-5D-5L is the number of response options available for each dimension. The EQ-5D-5L provides a more detailed and nuanced assessment of an individual’s health status compared to the EQ-5D-3L. Thus, for each dimension, respondents choose from five levels of severity: no problems, slight problems, moderate problems, severe problems, or extreme problems. By assigning digits to each level, a 5-digit number can represent the patient’s health state. To create a unified measure of health, the scores from these five dimensions were transformed into a single utility index using the R language and the eq5d package [39].

To our knowledge, this is the first study to build and optimize predictive models with AutoML for the purpose of predicting the risk of metabolic syndrome. In our research, we used two AutoML frameworks known for their efficiency in generating optimized models within a brief timeframe, namely H2O and Auto-sklearn, respectively.

H2O [40], a distributed machine learning platform, is specifically designed to scale efficiently, particularly with large data sets. By harnessing in-memory data compression, H2O can handle vast amounts of data, even with a modest cluster, making it highly versatile. The platform is adaptable, capable of operating on a local desktop with a small cluster or effortlessly scaling across multiple nodes using technologies such as Spark, an Amazon Elastic Compute Cloud (EC2) cluster, or Hadoop.

Auto-sklearn [41,42] stands out as a robust AutoML framework, constructed on the foundation of the scikit-learn [43] machine learning framework. Modified versions of Auto-sklearn 1.0 demonstrated exceptional performance by clinching the leading positions in both the inaugural and subsequent ChaLearn AutoML challenges. These challenges rigorously assessed AutoML systems within stringent time and memory limitations, demanding predictions to be generated in less than 20 min.

The advantage of building models with AutoML is that the hyperparameters of the models are optimized very efficiently, in a short time, with the possibility of imposing some time restrictions for the duration of the execution. Furthermore, the algorithms that yield the most accurate predictions on the given data set are automatically identified. The objective of the ML models was to differentiate between patients who were diagnosed with metabolic syndrome and those who were not, and to identify key predictors of metabolic syndrome.

The primary contributions of this study can be outlined as follows:

(1) Several predictive models were built using two state-of-the-art AutoML frameworks: H2O AutoML and Auto-sklearn.
(2) The best two models were selected, evaluated, and validated, comparing the prediction results through performance metrics.
(3) A SHAP wrapper specifically designed for Auto-sklearn models was implemented to obtain their corresponding SHAP values.
(4) SHAP was used to analyze machine learning models, to explain the predictions, and to highlight the most important predictive variables.

The aim of this observational study was to investigate the degree to which the general state of health and factors affecting oral health, particularly disruptive ones, are associated with metabolic syndrome, with a null hypothesis (H_0_) stating that a higher periodontitis stage [44] does not increase the incidence of metabolic syndrome. The significance of establishing these connections lies in the potential of implementation of a multidisciplinary treatment paradigm and suggests some procedures or techniques that could prevent/interrupt critical pathological links to provide positive results and enhance overall health.

Our best interpretable risk prediction models for metabolic syndrome have proven valuable in uncovering risk factors, identifying high-risk individuals, and serving as a methodological reference for the prevention and control of metabolic syndrome.

The rest of the paper is organized as follows: In Section 2, we provide a summary of related research in the field of prediction of metabolic syndrome/periodontal disease with ML models. Section 3 illustrates the proposed methodology, the collected data, and the performance metrics used in the evaluation of the prediction models. Section 4 presents the best ML models built and their hyperparameter values at the end of the optimization process. The values of the performance metrics of the prediction models are also presented. A substantial part of this section has been devoted to SHAP’s explanations of machine learning models. Finally, Section 5 and Section 6 conclude the study and offer insights into potential future directions.

## 2. Related Work

Table 1 provides a summary of the related research and refers to previous studies on predictions involving metabolic syndrome and/or periodontal disease, using ML models where they appear as the target variable or feature variable.

The study presented in [45] focuses on the development and testing of a prediction model for periodontal disease. Leveraging machine learning techniques and extensive electronic dental record data, the authors aimed to enhance accuracy in predicting periodontal disease. The research explores factors and patterns associated with periodontal conditions and aims to identify ways for more effective early detection and management of periodontal diseases.

In the retrospective cohort study published in [46], Yu et al. explore the use of machine learning models, specifically employing a Decision Tree Algorithm, for predicting metabolic syndrome. The study delves into the application of computational approaches to predict the likelihood of metabolic syndrome based on relevant data. The findings aim to enhance the understanding of metabolic syndrome prediction and potentially contribute to more personalized and proactive interventions in clinical settings.

The study conducted by Sghaireen et al., published in [47], employs a machine learning approach for diagnosing metabolic syndrome. Specifically, the authors utilize an explainable data-augmentation-based classification method. By integrating machine learning techniques into the diagnostic process, the research aims to enhance the accuracy and interpretability of metabolic syndrome diagnoses. The results provide insights into the potential of explainable data augmentation in improving the performance and transparency of classification models for metabolic syndrome.

In [48] is presented a machine learning-aided risk prediction model for metabolic syndrome, drawing insights from a comprehensive 3-year study. The authors employ machine learning techniques to predict the risk of metabolic syndrome, with the aim of offering valuable information for the development of proactive and personalized interventions in the context of metabolic syndrome.

Gutiérrez-Esparza et al. present a study in [49] where machine and deep learning methodologies are applied for the prediction of metabolic syndrome without the need for blood screening. The research explores innovative approaches to predict metabolic syndrome using computational techniques, potentially offering a non-invasive alternative to traditional blood tests.

In a retrospective cohort study published in [50], Zhang et al. employ machine learning techniques to predict the 4-year risk of metabolic syndrome in adults. The research focuses on leveraging computational methods for risk assessment over an extended period, offering insights into the potential of machine learning in predicting the development of metabolic syndrome.

In [51], the authors explore the development of metabolic syndrome prediction models by incorporating machine learning techniques and considering Sasang constitution types. The research investigates the integration of traditional medicine concepts, specifically Sasang constitution types, with modern computational methods for predicting metabolic syndrome. The findings provide insights into the potential synergy between machine learning and personalized medicine in predicting metabolic syndrome.

In their observational study published in [52], Monsarrat et al. introduce a systemic periodontal risk score developed through an innovative machine learning strategy. The research focuses on leveraging advanced computational methods to establish a comprehensive risk assessment for systemic health based on periodontal factors.

The research presented in [53] aims to develop and validate predictive models for periodontitis through a comprehensive evaluation of different machine learning techniques. By systematically comparing the performance of these algorithms, the study contributes valuable insights into the selection and optimization of models for predicting periodontitis.

The study [54] focuses on the development of a model that utilizes machine learning techniques to predict the risk of metabolic syndrome, emphasizing its potential in preventive healthcare. The findings highlight the role of machine learning in developing proactive strategies for maintaining metabolic health.

In a nested cross-sectional study published in [55], Pietropaoli et al. investigate the association between components of metabolic syndrome and gingival bleeding, with a specific focus on gender differences, especially in women. The research reveals a women-specific correlation between metabolic syndrome components and gingival bleeding. The findings contribute to a nuanced understanding of the relationship between systemic health and oral conditions, emphasizing gender-specific considerations in the context of metabolic syndrome and oral health.

A closer work to the one presented here is [56], where the authors investigate the relationship between periodontal status and metabolic syndrome in middle-aged Japanese individuals. The research explores potential associations between the two, providing insight on how periodontal health may relate to the presence of metabolic syndrome. The findings contribute to the understanding of the interplay between oral health and systemic conditions, particularly in the context of metabolic syndrome among the middle-aged Japanese population.

The authors of [57] utilize machine learning and statistical analyses to examine the interconnections among obstructive sleep apnea, metabolic dysfunction, and periodontitis. The research, based on the Dental, Oral, Medical Epidemiological (DOME) Big Data Study, employs advanced computational methods to explore complex relationships between these health conditions. The findings emphasize the potential of big data and machine learning in uncovering intricate health patterns.

In [58], the authors present a study where seven machine learning models are employed to predict the occurrence of metabolic syndrome. The research focuses on the application of computational approaches to forecast the likelihood of metabolic syndrome. By leveraging machine learning, the study contributes to the field of predictive medicine, offering insights into potential risk factors and facilitating proactive interventions for individuals at risk of developing metabolic syndrome.

Two other works closely related to the topic of our research are [59,60].

In their study [59], Nibali et al. investigate the relationship between left ventricular geometry and periodontitis in patients with metabolic syndrome. The research explores potential associations between periodontal health and cardiac parameters in individuals with metabolic syndrome. The findings contribute to the understanding of the systemic impact of periodontitis in the context of metabolic syndrome, particularly concerning left ventricular geometry. The study highlights the intricate links between oral health and cardiovascular parameters in individuals with metabolic syndrome.

In [60] is investigated the association between periodontitis and its severity levels with the triglyceride/high-density lipoprotein cholesterol (TG/HDL-C) ratio. The study explores potential connections between periodontal health and the TG/HDL-C ratio, providing insights into the systemic impact of periodontitis on metabolic parameters. The findings underscore the relevance of oral health in relation to lipid metabolism and cardiovascular risk factors, emphasizing the association between periodontal status and the TG/HDL-C ratio.

Compared to a previous study [56], which is based on multivariate logistic regression to achieve relatively similar objectives using only two predictors (periodontal probing depth and clinical attachment level), we have expanded the set of feature variables, and we built optimized ML models to predict the risk of metabolic syndrome in individuals with periodontal disease. Conventional regression techniques used in [56,59,60] are straightforward to apply but face challenges when handling multifactor effects and nonlinear relationships in data mapping. Complex ML models obtained with our proposed methodology have the capacity to learn intricate patterns and representations from the input features and can capture non-linear relationships in the data, which may be missed by linear models like logistic regression.

We noticed that no previous research had been conducted in employment of the AutoML technology for obtaining highly optimized ML models. Our approach can leverage pre-trained models on large data sets for transfer learning (technique implicitly used by AutoML frameworks). This is especially useful when we have small data sets. In addition, another advantage is the automatic selection of the best algorithm and its fast training, problems that papers [53,58] addressed through comprehensive evaluation of different machine learning algorithms. The respective approach requires a lot of time, high expertise, and there is no guarantee of obtaining the best solution.

Our suggested approach, based on AutoML, provides the opportunity to completely automate the maintenance of prediction models, ensuring their high accuracy by automatically regenerating or retraining as needed.

To increase the transparency and acceptance of our ML models and to strengthen confidence in the results obtained, we made use of permutation explainer implemented in the SHAP framework. The same framework was used to explain the predictions in papers [45,47,48,52].

## 3. Materials and Methods

### 3.1. Efficient Model Development with AutoML Frameworks

Machine learning (ML) has made impressive strides in recent years. However, the conventional process of developing ML models is resource-intensive, demanding substantial domain expertise and time to create and evaluate numerous models for comparison [61].

Automated machine learning, also referred to as automated ML or AutoML, is the process of automating the repetitive and time-consuming tasks involved in developing machine learning models. AutoML offers techniques and workflows that enable the creation of ML models at a large scale, with increased efficiency and productivity, all while maintaining high model quality.

The automated machine learning (AutoML) approach typically involves several key steps to provide an optimized model ready for deployment:

1. **Model Selection**: Automatically selecting an appropriate machine learning model or algorithm based on the problem type (e.g., classification, regression) and data set characteristics.
2. **Feature Selection**: Choosing a subset of relevant features or variables from a larger set of available features, to improve the overall efficiency of the machine learning pipeline (improve model interpretability and reduce computational complexity).
3. **Hyperparameter Tuning**: Optimizing the hyperparameters of the selected model(s) to improve their performance.
4. **Model Training**: Training the selected model(s) on the training data set using the optimized hyperparameters.
5. **Validation**: Evaluating model performance on the validation data set to ensure that it meets predefined criteria, such as accuracy or F1 score. If the model does not meet the criteria, it may return to hyperparameter tuning or model selection steps.

**Figure 1:**
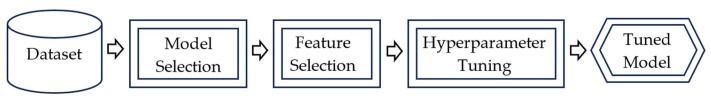
Architecture of a generic AutoML pipeline.

An AutoML pipeline is a series of automated and interconnected processes designed to streamline the end-to-end machine learning workflow. The primary goal of an AutoML pipeline is to automate the machine learning process. A generic AutoML pipeline is shown in Figure 1.

In this paper, we propose to create predictive models with two state-of-the-art AutoML frameworks: H2O AutoML [62] and Auto-sklearn [63].

H2O is a distributed machine learning platform designed to handle extensive data sets efficiently. It offers application programming interfaces (APIs) for popular programming languages such as R, Python, and Java, making it accessible and versatile for a wide range of data science and machine learning tasks.

H2O’s AutoML framework offers a set of classes and functions designed to streamline various modeling tasks with just a few lines of code. This tool is particularly useful for automating the entire machine learning workflow, encompassing the automatic training and fine-tuning of multiple models within a predefined time limit specified by the user [40]. After training the base models, the H2O’s Stacked Ensemble algorithm is used to train two Stacked Ensemble models. By default, H2O uses a Super Learner algorithm [64] to train the metalearner in the Stacked Ensemble, using the *k*-fold cross-validated predictions from the base learners.

In the following sections, we will leverage H2O AutoML to automatically select and fine-tune models. Subsequently, the best-performing model, often referred to as the “leader”, will be utilized for making predictions.

Auto-sklearn is a robust automated machine learning (AutoML) framework that utilizes scikit-learn as its underlying machine learning framework [43]. This framework streamlines the process of automating machine learning tasks while leveraging the capabilities and versatility of scikit-learn for model development and evaluation.

Auto-sklearn uses the random-forest-based Bayesian optimization method SMAC (Sequential Model-based Algorithm Configuration) [65] to solve the Combined Algorithm Selection and Hyperparameter optimization (CASH) problem [41,42].

After building predictive models, two equally important steps follow: model validation and model explanation.

### 3.2. Model-Agnostic Explainability with SHAP Method

Model explanation involves the use of specific tools to inspect the structure of the model, highlight the importance of features, and interpret the predictions of ML models. For this purpose, in this paper we used the SHAP (SHapley Additive exPlanations) framework, proposed by Lundberg and Lee [35] as a unified approach, designed to provide a comprehensive explanation of the results produced by any machine learning model.

In the process of explaining a machine learning model, Shapley values can be thought of as a measure of the importance of each individual input feature’s contribution to the model’s predicted values. They help to understand how each feature influences the model’s output and provide valuable insight into its decision-making process. In the following, we present the concepts and procedure underlying the calculation of SHAP values.

One way to explain a prediction is to attribute the model prediction to each feature, considering the interactions and dependencies between features.

In game theory, a coalitional game involves a set of players who can form coalitions (groups) and receive certain payoffs. The Shapley value is a solution concept that allocates a fair share of the total payoff to each player based on their marginal contributions when joining different coalitions.

In the context of machine learning, each feature of an instance is treated as a “player” in a game, and the model prediction is the total payoff. Shapley values help determine how to fairly distribute this total payoff among the individual features.

The Shapley value for a feature measures the average marginal contribution of that feature across all possible combinations of features and offers a transparent and interpretable way to understand the impact of the feature on a specific prediction, considering the interactions and dependencies with other features.

Shapley values provide insights into the relative importance of different features in making a particular prediction, promoting transparency and understanding in complex machine learning models.

A cooperative game is defined as a function $v:2^{d}\to R$ that for each coalition (subset) S⊆D return a value v(S)∈R, where D={1,…,d) represents a set of players [66]. We can consider that v(S) represents the profit generated by the coalition *S* of players. The payoff of each player i∈S is equal to his contribution to the realization of the profit and will be denoted by $\phi_{i}(v)$. For a fairly assessment of these individual contributions, the following goals are imposed [67]:

1. (Efficiency) The contributions should add up to the difference between the profit generated by all players and the profit obtained without any player:
  $$\sum_{i=1}^{d}\phi_{i}(v)=v(D)-v(\emptyset ).$$
2. (Symmetry) If two players are interchangeable (the impact in the generated profit is the same), it follows that their individual contributions are identical, or as follows: $v(S\cup \{i\})=v(S\cup \{j\})\;\;for\;\;all\;\;S\Rightarrow \phi_{i}(v)=\phi_{j}(v)$.
3. (Dummy) If a player has no impact in generating profit, then his contribution is zero, or as follows: $v(S\cup \{i\})=v(S)\;\;for\;\;all\;\;S\Rightarrow \phi_{i}(v)=0$.
4. (Monotonicity) If the marginal contribution to the profit generated in game v by player i by joining any coalition S is greater than that obtained in game $v^{\prime }$, then the contribution of the player i in v is greater than in $v^{\prime }$, or as follows: $v(S\cup \{i\})-v(S)\geq v^{\prime }(S\cup \{i\})\;-v^{\prime }(S)\;for\;\;all\;\;S\Rightarrow \phi_{i}(v)\geq \phi_{i}(v^{\prime })$.
5. (Linearity) If the game v is viewed as a linear combination of games $v_{1},v_{2},\ldots ,v_{k}$, or $v=c_{1}v_{1}+c_{2}v_{2}+\ldots +c_{k}v_{k}$, then the contribution of each player i in the game v is expressed as follows: $\phi_{i}\left(v\right)=c_{1}\phi_{i}\left(v_{1}\right)+c_{2}\phi_{i}\left(v_{2}\right)+\ldots +c_{k}\phi_{i}\left(v_{k}\right)$, i∈D.

In [68], it has been shown that for any cooperative game v, the values $\phi_{i}(v)$ (the Shapley values of v) calculated with the formula

$$\phi_{i}(v)=\frac{1}{d}{\sum_{S\subseteq D\backslash \{i\}}\left(\begin{array}{l} d-1\\ \;\;\;|S| \end{array}\right)}^{-1}[v(S\cup \{i\})-v(S)]=\sum_{S\subseteq D\backslash \{i\}}\frac{|S|\;\;!\;\;\;\;(d-|S|-1)!}{d!}\;\;\;[v(S\cup \{i\})-v(S)],\;i\in D$$

are the only values that satisfy properties 1–5.

The primary obstacle in utilizing Shapley values lies in achieving computational efficiency during their calculation. To compute the precise $\phi_{j}$ Shapley value, it is necessary to assess all potential combinations (sets) of feature values both with and without the j-th feature. However, as the number of features grows, the exact solution becomes challenging due to the exponential increase in possible coalitions. This exponential increase in complexity poses a significant challenge, making it impractical to calculate Shapley values efficiently, especially when dealing with a large number of features.

SHAP, introduced in [35], is an interpretability method that explains individual predictions by assigning attribution scores to each feature using approximate Shapley values. Next, we will focus on KernelSHAP, a kernel-based estimation approach for Shapley values, which can be used for any ML model [35].

In the field of supervised learning, we consider a scenario where a model denoted by *f* is used to predict the outcome variable *Y*, given as input *X*, where *X* is a set or individual features (*X*_1_, *X*_2_, …, *X*_d_). In the following, uppercase symbols (e.g., *X*) are employed to represent random variables, while lowercase symbols (e.g., *x*) are used to denote specific values.

For a subset S⊆D, the restricted model $f_{S}$ is defined as follows [67]:


$$f_{S}(x_{S})=E\left[f(X)|X_{S}=x_{S}\right]$$

There are two special cases: S=∅ and S=D. These correspond to the mean prediction $f_{\emptyset }(x\emptyset )=E[f(x)]$ and the full model prediction $f_{D}(x)=f(x)$, respectively.

Each subset *S* (of features) can be associated with a binary vector $z\in {\left\{0,1\right\}}^{d}$ such as $S=\{i:z_{i}=1\}$ (1 = feature present in coalition, 0 = feature absent) [66]. If we denote by m<d the maximum coalition size, $z^{\prime }\in {\left\{0,1\right\}}^{m}$ represents a coalition vector of selected features $M=\{s_{1},s_{2},\ldots ,s_{m}\}\subset D$.

SHAP aims to explain the prediction of a specific instance $x=(x_{1},x_{2},\ldots ,x_{d})$ by determining the contribution of each feature to that prediction. SHAP defines the explanation as follows:

(1) $$e(z^{\prime })=\phi_{0}+\sum_{j=1}^{m}\phi_{j}{z^{\prime }}_{j}$$

where e is the explanation model, $\phi_{0}=E\left[f(X)\right]$ and $\phi_{j}$ is the Shapley value for the feature j. Coalition vectors maps to the original inputs through a mapping function $h_{x}:{\left\{0,1\right\}}^{m}\to {\mathbb{R}}^{d}$ defined as follows:

$$h_{x}(z^{\prime })=z=(z_{1},z_{2},\ldots ,z_{d})\;\;\;\mathrm{where}\;z_{i}=\left\{\begin{array}{l} x_{i},\;\;if\;\;i\in D\backslash M\\ x_{i},\;\;if\;\;i=s_{k}\in M\;\;and\;\;{z^{\prime }}_{k}=1\\ random\;\;value\;\;of\;feature\;\;X_{s_{k}},\;\;if\;\;i=s_{k}\in M\;\;and\;\;{z^{\prime }}_{k}=0 \end{array}\right.$$

This implies that $h_{x}$ treats the absence of a feature value by substituting it with a random value from the existing data for that feature.

KernelSHAP is a linear regression-based approximation method, described by the following procedure [69]:

1. Generate *K* sample coalitions: ${z^{\prime }}_{k}\in {\left\{0,1\right\}}^{m},\;\;\;1\leq k\leq K$. These compose the data set for the regression model.
2. Get prediction for each ${z^{\prime }}_{k}$, by mapping it into the original feature space and then applying the model *f*: $f(h_{x}({z^{\prime }}_{k}))$
3. Compute the weight for each ${z^{\prime }}_{k}$ with the SHAP kernel, defined by the following formula:
  $\pi_{x}({z^{\prime }}_{k})=\frac{m-1}{\left(\begin{array}{l} \;\;\;\;\;m\\ \left|{z^{\prime }}_{k}\right| \end{array}\right)|{z^{\prime }}_{k}|(m-|{z^{\prime }}_{k}|)}$, where $\left|{z^{\prime }}_{k}\right|$ is the number of present features in the coalition.
4. Train the linear regression model (1) by minimizing the following loss function:
  $$L(f,e,\pi_{x})=\sum_{z^{\prime }\in Z}[f(h_{x}(z^{\prime }))-e(z^{\prime }){]}^{2}\pi_{x}(z^{\prime })$$
  where $Z=\{{z^{\prime }}_{1},{z^{\prime }}_{2},\ldots ,{z^{\prime }}_{K}\}$ is the training data.
5. Return approximate Shapley values $\phi_{j}$ (coefficients of the linear regression model).

SHAP values are a powerful tool for understanding and interpreting the output of machine learning models, offering insights into the contribution of each feature to individual predictions. For example, they provide a nuanced understanding of the importance of each feature in making predictions and the SHAP framework considers that features with large absolute Shapley values are important. For a feature *X_j_*, its importance is computed with formula

$$I_{j}=\frac{1}{n}\sum_{i=1}^{n}\left|\phi_{j}^{\left(i\right)}\right|$$

where *n* is the number of records in the data set.

The variable importance plot, presented in Section 4.2.1, offers a visual representation of feature importance. It lists feature variables in descending order of mean SHAP values, providing a graphic illustration of their significance.

Because our approach relies on AutoML, where the prediction algorithm is automatically selected, it is crucial that the calculation of SHAP values is conducted using a model-agnostic method, such as KernelSHAP.

The SHAP framework [70] includes a universal SHAP explainer for any ML algorithm, provided by the KernelExplainer class, which implements the procedure described above.

We used the model-agnostic KernelExplainer class with a wrapper for H2O models, developed by [71]. In order to have the same approach for Auto-sklearn, we implemented a wrapper for this framework as well, the code being presented in Table 2.

Thus, in this unitary approach, the function of model’s predictions represents the probability of predicting the *True* class (the existence of metabolic syndrome) for each instance in the data set.

### 3.3. Study Design

The study was conducted from May 2018 to December 2019 and involved patients hospitalized in the Cardiology and Diabetes clinical departments of the Sibiu County Emergency Clinical Hospital. It received approval under reference number 10948/2018 from the Ethics Committee. Our study exclusively enrolled patients who read and signed the informed consent form.

The study group comprised 296 participants, ranging in age from 45 to 79 years, with an average age of 66.1. Among these participants, 172 were female, with an average age of 65.7, and 124 were male, with an average age of 66.5.

The medical data of the hospitalized patients were retrieved from the clinical observation sheets, while information regarding their dental health was acquired through dental consultations conducted at the Dental Clinic of the Sibiu County Emergency Clinical Hospital. From the collected data, 168 patients could be categorized based on the criteria established in the existing literature [29] as falling within the clinical spectrum of metabolic syndrome.

The patients were examined sitting on the dental chair, illuminated by a sciatica lamp, and the examination was performed by a single doctor, to ensure the consistency of the results and to avoid their variation.

During the examination, a plane mirror and the CPI (Community Periodontal Index) periodontal probe with the abutment tip were used, according to the guidelines provided by the World Health Organization (WHO) [29], which recommends the use of a CPI probe with a 0.5-mm ball tip.

We recorded the following clinical data: the depth of the gingival groove, measured in millimeters, at three locations on the vestibular surface and three additional locations on the oral surface of the teeth designated as per the Ramfjord index, including teeth 16, 11, 26, 36, 41, and 46. In cases where teeth were missing, measurements were conducted on homologous teeth within the same sextant [29].

We recorded Clinical Attachment Loss (CAL) as the distance between the attachment level and the enamel-cement junction. Additionally, we noted instances of spontaneous bleeding from the gingival sulcus. The presence of bacterial plaque and tartar was also measured in millimeters. Information regarding dental caries, fillings, and teeth extracted due to dental caries-related complications was recorded in relation to each tooth’s condition. We utilized the collected data to compute the following indices: the Community Periodontal Index (CPI) and the DMFT index (Decay Missing Filling Tooth).

After the dental consultation, the patients were questioned in the following respects: the number of teeth brushing performed daily, the preventive dental control, weekly physical activities more than 30 min/day minimum of three times a week. The patients also responded to a standard EQ-5D-5L questionnaire.

To investigate the risk factors contributing to the clinical development of metabolic syndrome, our approach involves constructing predictive models that incorporate various independent variables representing lifestyle characteristics.

### 3.4. Data Set

The data set, consisting of the 296 subjects under analysis, was split into two distinct subsets: the training set, which constituted 70% of the data, and the test set, which comprised the remaining 30%.

The ML model incorporates the following variables:

- Dependent variable (target): Metabolic syndrome.
- Independent variables (feature variables): DMFT (Decayed, Missing due to caries, and Filled Teeth), CPI (Community Periodontal Index), Periodontal pockets depth, Gingival bleeding, Daily tooth brushing, Dental control, Gingival attachment loss, CV (Cardiovascular) risk, Carotid atherosclerosis, and EQ-5D-5L score.

In the data preprocessing stage, certain categorical variables (listed in Table 3) were assigned numerical values.

**Figure 2:**
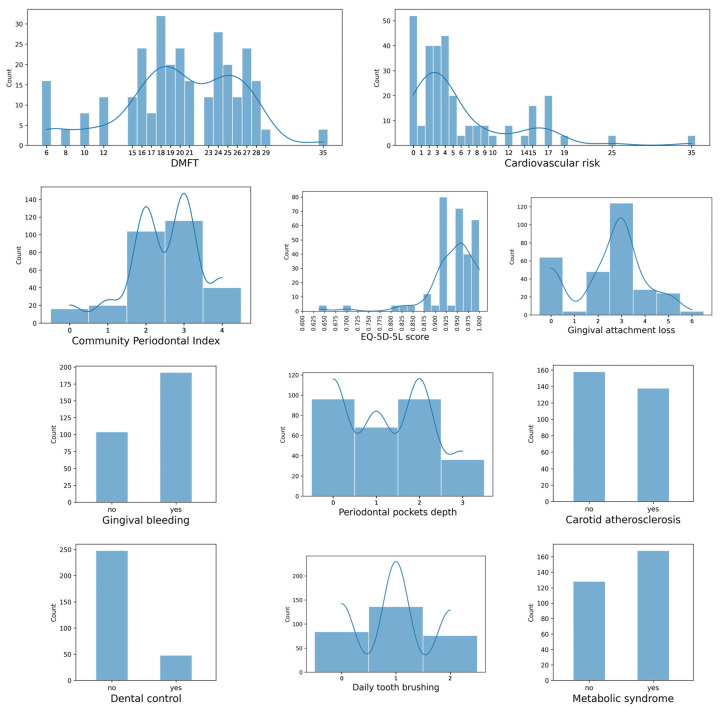
Graphical overview of the data set.

Figure 2 provides a graphical overview of the data set. The histograms show the frequencies of the ML model variables. In addition to the histogram, in some charts a smoothed curve representing the estimated probability density function of the data using Kernel Density Estimation is overlaid.

### 3.5. Performance Evaluation

The validation of the model involves assessing its performance, with a primary focus on the accuracy of the predictions it generates. This evaluation helps determine how well the model is able to make correct predictions and is a crucial step in ensuring the model’s reliability and effectiveness.

**Figure 3:**
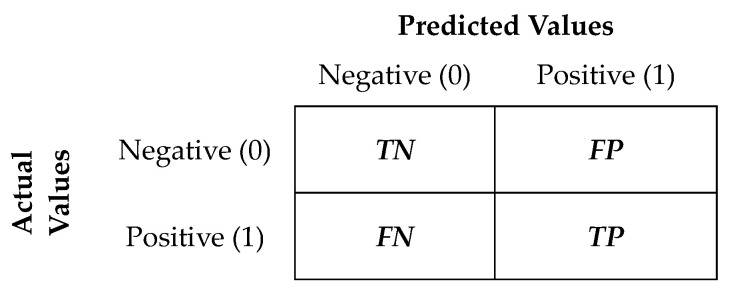
Confusion Matrix for a binary classification data set.

The classification models were evaluated using various performance metrics based on Confusion Matrix. In case of binary classification, the Confusion Matrix is represented as in Figure 3, and the interpretation of its elements can be found in Table 4.

The values of the elements in the confusion matrix were utilized to compute the following classification metrics (Table 5), which are used to evaluate how well a model performs in categorizing data into different classes or categories.

Accuracy represents the proportion of correctly classified instances out of the total number of instances. Precision measures the accuracy of positive predictions (it is the ratio of true positive predictions to the total positive predictions). The Recall metric measures the model’s ability to correctly identify positive instances (it is the ratio of true positive predictions to the total actual positive instances). The F1 score is the harmonic mean of precision and recall. It balances precision and recall and is especially useful when dealing with imbalanced data sets. The Specificity metric (or true negative rate) measures the model’s ability to correctly identify negative instances (it is the proportion of true negatives out of all actual negatives instances). Balanced accuracy is calculated as the arithmetic mean of Recall (true positive rate) and Specificity (true negative rate). It provides a more comprehensive evaluation of a classification model’s performance, particularly when dealing with imbalanced data sets.

## 4. Results

### 4.1. Prediction Models

Two AutoML frameworks (H2O AutoML and Auto-sklearn) were utilized to automatically search for the best prediction models tailored to our data set. For the generation and optimization of the models, the execution time was set to 15 min.

The best models provided by each of the two frameworks are shown in Table 6, and the values of the performance metrics of the respective prediction models are presented in Table 7.

The Distributed Random Forest (DRF) model averages multiple decision trees, each created on different random samples of rows and columns. It is capable of handling non-linear relationships and offers insights into the significance of each predictor within the model. These characteristics collectively make it one of the most robust algorithms, particularly suitable for dealing with noisy or complex data sets.

Random Forest (RF) builds a set of decision trees that work as an ensemble. Each tree is developed from a sample from the training data. When developing individual trees, an arbitrary subset of attributes is used (hence the term “random”), from which the best attribute is selected for splitting. The final model is based on the majority vote of the set of individually grown trees that are part of the forest.

Gradient Boosting Machine (GBM) is a forward learning ensemble method that leverages the concept of boosting to build an ensemble of decision trees. Its fundamental principle is to achieve accurate predictions by iteratively refining approximations. The learning process is sequential, where each tree is constructed to correct the errors or residuals of the previous ones. This is achieved by assigning higher weights to the misclassified or poorly predicted instances.

XGBoost (eXtreme Gradient Boosting) is an advanced and highly efficient implementation of a gradient boosting algorithm. One of the key strengths of XGBoost is its effective regularization techniques, which help control overfitting and contribute to its superior performance. The algorithm is robust when it comes to handling irregularities in data (it can handle missing values, outliers, and noisy data effectively). It leverages parallel computation, meaning that it can train multiple decision trees concurrently to find the final prediction. The XGBoost model requires parameter tuning to improve and fully exploit its advantages over other algorithms, and we used the H2O framework for this purpose.

The MLP (Multi-layer Perceptron) classifier is a type of neural network that trains iteratively. In each iteration, it computes the partial derivatives of the loss function with respect to the model parameters, which are then used to update these parameters. This iterative process allows the model to learn and improve its predictions. It incorporates a regularization term in the loss function to prevent overfitting. The Auto-sklearn model is optimizing the log-loss function, which is a common evaluation metric for binary classification models. It measures the performance of a model by quantifying the dissimilarity between predicted probabilities and actual class labels.

The ExtraTrees classifier is a machine learning algorithm that serves as a meta-estimator. It operates by fitting multiple randomized decision trees, often referred to as “extra-trees”, on different sub-samples of the data set. The primary goal of using this ensemble approach is to improve predictive accuracy while also mitigating the risk of overfitting.

All models were evaluated using the performance metrics presented in Table 5. The results, shown in Table 7, were achieved by evaluating the machine learning models using the test data set as input, and allowed us to assess how well the models generalize to unseen data and make predictions in a real-world context.

It is observed that the classification errors of the H2O models (apart from XGBoost) are of the *FN* (False Negative) type. In the case of Auto-sklearm models, all models present some *FP* (False Positive) classification errors.

For the interpretability of the prediction models, we will restrict the analysis to the best models provided by each of the two frameworks, namely XGBoost (H2O) and RF (Auto-sklearn).

**Figure 4:**
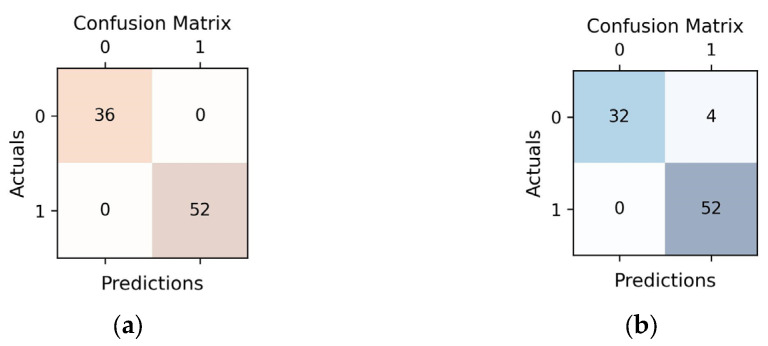
Confusion matrices representing predictions vs. actuals on test data for each of the two prediction models (**a**) H2O AutoML (XGBoost); (**b**) Auto-sklearn (RF).

Figure 4 illustrates that the best H2O model achieved perfect classification, correctly identifying all cases. In contrast, the best Auto-sklearn model, while generally effective, made incorrect classifications for 4 cases (*FP*). This suggests that the H2O model had a higher overall accuracy in classifying the data, whereas the Auto-sklearn model had a slightly lower accuracy with a few misclassifications.

In our case, we can say that we have a winning model, but in general different models may excel in specific metrics, highlighting the importance of considering multiple evaluation criteria when selecting the most suitable model for predictive analytics.

### 4.2. Explainability of Prediction Models Using SHAP Framework

The primary challenge in comprehending many machine learning models is their “black box” nature [72]. In numerous applications, understanding the reasons behind a model’s specific prediction can be just as critical as the accuracy of the prediction itself. After training a model, it becomes essential to understand the effects and interactions of the attributes that contribute to the classification process.

The KernelExplainer class from the SHAP framework, which implements the Kernel SHAP method, was used to interpret the predictions, assigning to each feature variable of each instance the SHAP value (importance value) for a given metabolic syndrome score prediction.

SHAP values offer two significant advantages:

- Global interpretability—the SHAP values provide a comprehensive view of how each predictor contributes to the target variable, offering insights into both positive and negative influences. This allows understanding the overall impact of each feature on the model’s predictions.
  Local interpretability—each observation is assigned its own set of SHAP values. Thus, one can explain why a case receives its prediction and the contributions of the predictors.
- SHAP does not have direct support for H2O models.

#### 4.2.1. Global Interpretability

Collective SHAP values offer insights into how much each predictor contributes, whether positively or negatively, to the target variable.

For preliminary data exploration, we created a correlation matrix for the model variables (based on Pearson Correlation Coefficient). For reasons of space, the set of feature variables was divided into two equal parts, and the target variable was kept in both matrices.

**Figure 5:**
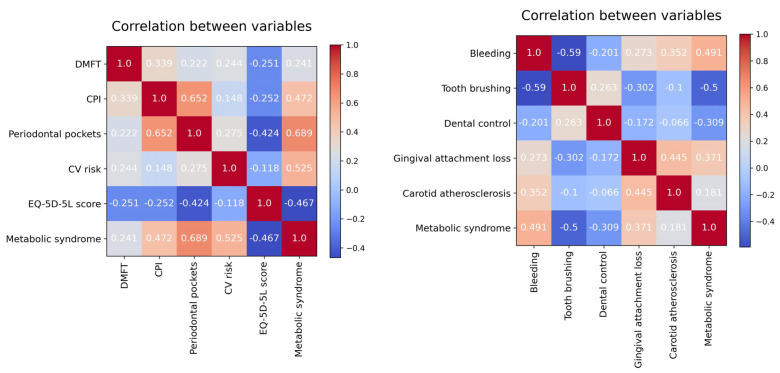
Correlation matrix for the model variables.

Looking at the correlation matrices from Figure 5, it seems that Metabolic syndrome has the following characteristics:

- a strong positive correlation with Periodontal pockets (depth) and CV risk;
- a moderate positive correlation with CPI, (gingival) Bleeding, and Gingival attachment loss;
- a moderate negative correlation with EQ-5D-5L score, (daily) Tooth brushing, and Dental control.

Metabolic syndrome is not significant linearly correlated with either DMFT or Carotid atherosclerosis.

In the following, for each feature, the average SHAP value for all observations was calculated. Specifically, the average of the absolute values was considered because positive and negative values must be avoided to offset each other.

Features that have made substantial positive or negative contributions to the model’s predictions will indeed have large mean SHAP values. These large mean SHAP values indicate that these features have had a significant and influential role in shaping the model’s outputs. In essence, they are the features that the model relies on most heavily when making its predictions, whether positively or negatively.

**Figure 6:**
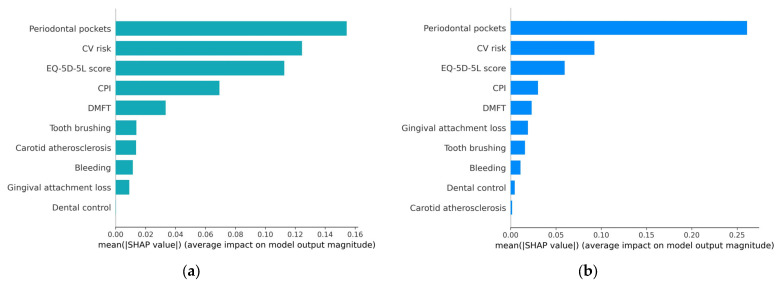
Variable importance plot: (**a**) H2O AutoML (XGBoost); (**b**) Auto-sklearn (RF).

In the variable importance plot, presented in Figure 6, feature variables are listed in descending order of the mean SHAP values, with the most significant variables at the top. These top variables contribute more to the model’s performance than the ones at the bottom, indicating their high predictive power. This visualization helps identify which features have the most substantial impact on the model’s predictions, aiding in feature selection, model understanding, and decision-making.

From the graphical representation, it is evident that periodontal pockets have the most substantial influence on the occurrence of metabolic syndrome. Following that, in descending order of importance are CV risk, EQ-5D-5L score, CPI, and DMFT. The other, less important feature variables have a different impact in the two prediction models (except for Bleeding).

A different perspective on the DMFT variable can be seen in Figure 5 and Figure 6, respectively. Although DMFT is not significantly linearly correlated with metabolic syndrome, it has an important role in the prediction model. The explanation is that the SHAP values provide the contribution of a feature to a prediction, which can be significant, even if there is no linear correlation between the feature and the target variable.

This ranking of variables by their influence on the outcome can provide valuable insights into the factors that most strongly contribute to the presence of metabolic syndrome.

**Figure 7:**
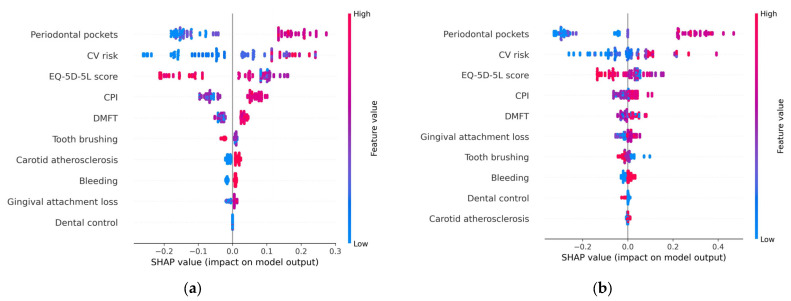
Summary (beeswarm) plot: (**a**) H2O AutoML (XGBoost); (**b**) Auto-sklearn (RF).

The summary plot (beeswarm) depicted in Figure 7 provides a visual representation of how these risk factors collectively contribute to the determination of metabolic syndrome, indicating their relative importance in the overall picture. This illustration helps convey how these factors interact and influence the syndrome’s occurrence, offering a clearer understanding of their combined impact.

Each point on the chart represents a SHAP value associated with a particular prediction and a specific feature.

The summary plot visualizes all SHAP values and is designed to display an information-dense summary of how the features variables impact the model’s output. It is one of the most important SHAP charts which can be used to highlight important relationships of the predictors with the target variable, considering the following aspects:

- **Feature importance**: Variables are ranked in descending order, based on their significance or importance, just like in the variable importance plot.
- **Impact**: In the beeswarm chart, the *x*-axis represents the SHAP values, computed for each feature of each record in the data set. If a SHAP value is on the right side of the plot, it corresponds to a positive impact on the prediction, leading the model to predict 1 (metabolic syndrome). Conversely, if a SHAP value is on the left side of the plot, it corresponds to a lower prediction or outcome which causes the model to predict 0 (absence of metabolic syndrome).
- **Value**: colors are used to indicate whether a feature variable’s value is relatively high (shade close to red) or low (shade close to blue) for a specific observation.
- **Correlation***:* The summary plot shows the positive and negative relationships of the predictors with the target variable. The position of the point along the horizontal axis shows how the feature’s value for that observation affects the prediction (higher or lower). Thus, a *high* depth of periodontal pockets has a *positive* association with metabolic syndrome. The “high” comes from the red color (which corresponds to high values of the variable), and the “positive” impact is shown on the *x*-axis (the SHAP value is on the right side of the plot). Similarly, we will say the “EQ-5D-5L” is negatively correlated with metabolic syndrome (target variable). From the charts represented in Figure 7, it can be concluded that high values (red dots) of the variables CV risk, CPI, DMFT, Carotid atherosclerosis, Gingival attachment loss, and (gingival) Bleeding are associated with positive SHAP values, so they correspond to an increased probability of occurrence of metabolic syndrome. High values of the variables (daily) Tooth brushing, Dental control, and EQ-5D-5L (represented in the charts by red dots) correspond to small (negative) SHAP values when compared to the low values of these feature variables. This suggests that as these variables increase (e.g., higher levels of tooth brushing, better dental control, or higher EQ-5D-5L scores), the probability of the occurrence of metabolic syndrome decreases.

**Figure 8:**
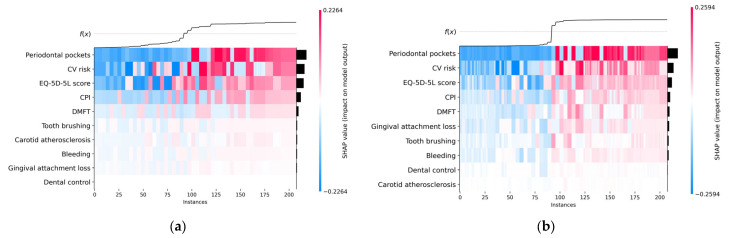
Heatmap plot: (**a**) H2O AutoML (XGBoost); (**b**) Auto-sklearn (RF).

The heatmap plot presented in Figure 8 contains important information. The black horizontal bars on the right rank the variables from most important to least important, the order being the same as in the beeswarm chart. The color of the line above each instance is used to represent the SHAP value of a particular feature for that instance. The color scale ranges from low (blue color) to high (red color). The heatmap plot highlights patterns between SHAP values and instance groups; therefore, the order of the instances is important for finding patterns. We opted for an ordering based on the sum of SHAP values over all features.

The *f(x)* curve on the top of the heatmap charts shown in Figure 8 represents the model’s predictions. Thus, for each instance *x*, *f(x)* represents the probability of predicting the *True* class (the existence of metabolic syndrome) for *x.*

Analyzing the heatmap plots, it can be seen that the observations were arranged so that the colors clustered together.

In the context of global interpretability (relations of predictors with the target variable), the SHAP values of the most important features (Periodontal pockets, CV risk, EQ-5D-5L score, CPI, and DMFT) have the greatest impact in the predictions made for both ML models (H2O and Auto-sklearn).

It can be seen from Figure 8 that for small SHAP values (blue color) we have *f(x)* = 0 (*False*), which implies the absence of metabolic syndrome, and for high SHAP values (red color) of these features we have *f(x)* = 1 (*True*), so those instances are associated with metabolic syndrome.

It should be noted that a high SHAP value is not necessarily correlated with a high value of the respective feature variable.

**Figure 9:**
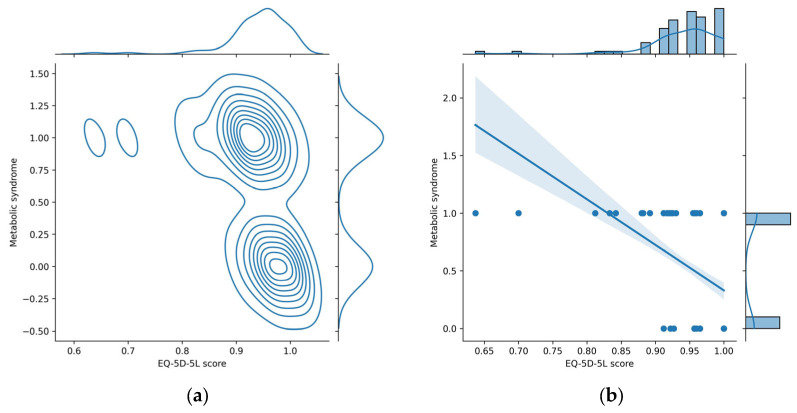
Joint plots for *Metabolic syndrome* and *EQ-5D-5L score* variables. (**a**) Kernel density estimate plot; (**b**) regression plot.

We used a jointplot data visualization tool to build graphical representations (depicted in Figure 9) that show the relationship between *Metabolic syndrome* and *EQ-5D-5L score* variables, along with their univariate distributions.

The jointplot with a kernel density estimate (KDE) (Figure 9a) displays in the main panel a scatter plot of the two variables against each other (each point on the scatter plot represents a data point with values for both variables), but instead of just showing individual points, KDE jointplot overlays contour lines to represent the estimated density of points in different regions, providing a smooth representation of the joint distribution. This is particularly useful for identifying patterns and concentrations in the data. The top and right panels display smoothed histograms (kernel density plots) for each variable, which provide a smooth representation of the distribution of each variable, capturing the overall shape and characteristics.

The jointplot with a regression (Figure 9b) provides a visualization of the relationship between two numerical variables, along with a linear regression line and additional statistical information. This type of jointplot adds a linear regression line to the scatter plot, which represents the best-fitting line through the data points and is useful for understanding the overall trend or direction of the linear relationship between the variables. The shaded area around the regression line represents the confidence interval for the regression estimate. This interval indicates the range within which the true regression line is likely to fall. The top and right panels display histograms representing the marginal distributions of each variable, which provide insights into the individual characteristics of each variable. The negative slope of the regression line from Figure 9b suggests a negative correlation between *EQ-5D-5L* and *Metabolic syndrome*: low values of *EQ-5D-5L score* are associated with the increased value for *Metabolic Syndrome* (1); thus, the occurrence of metabolic syndrome is very likely for low values of *EQ-5D-5L score*.

**Figure 10:**
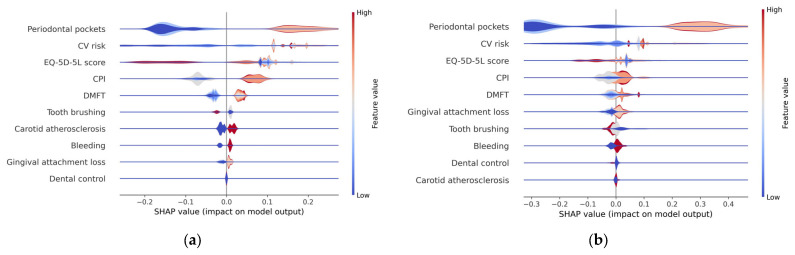
Layered violin plot (**a**) H2O AutoML (XGBoost); (**b**) Auto-sklearn (RF).

From Figure 9 it can be seen that low *EQ-5D-5L* values (<0.85) are associated with the presence of metabolic syndrome (*Metabolic syndrome* = 1). In the beeswarm chart (Figure 7), these low values correspond to high SHAP values, represented in the heatmap plot (Figure 8) with red color. In conclusion, in the heatmap plot, for the *EQ-5D-5L score* feature variable, large sharp values correspond to small values of the respective variable. This conclusion is clearly found in the layered violin chart below. In this chart, represented in Figure 10, the variation of the feature values at each SHAP value is clearer. From the graphic representation, it is found that small values of the *EQ-5D-5L score* feature variable (represented by blue color) correspond to relatively high SHAP values (located to the right on the *x*-axis).

#### 4.2.2. Local Interpretability

Local explainability aims to provide insights into the underlying factors that influence a particular prediction. It focuses on explaining the decision-making process for an individual instance within a model.

Given that each instance in the data set is associated with its own set of SHAP values, it becomes possible to explain why a specific instance receives a particular prediction and the individual contributions of the predictors in that context. This greatly increases the transparency of the predictive model and significantly increases the confidence in the predictions made.

The waterfall graph provides a comprehensive understanding of the stepwise process leading to the prediction results. It allows for a visual representation of how various factors and predictors contribute incrementally to the final prediction.

**Figure 11:**
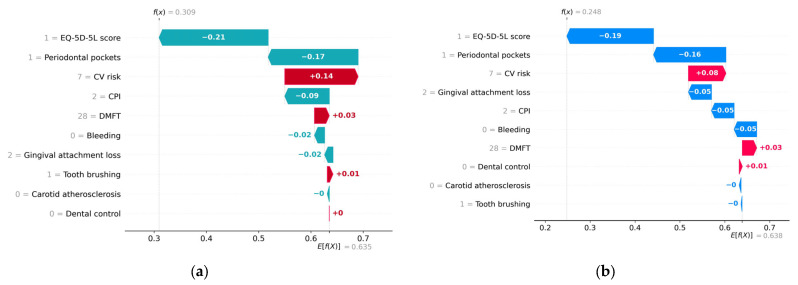
Waterfall plot for a healthy subject without metabolic syndrome. (**a**) H2O AutoML (XGBoost), predicted probability = 0.31; (**b**) Auto-sklearn (RF), predicted probability = 0.25.

Figure 11 provides an example of a healthy subject predicted to have a probability of 0.31 of metabolic syndrome according to the H2O model and a probability of 0.25 according to the Auto-sklearn model.

On the *x*-axis, the *E*[*f(x)*] represents the average predicted values across the testing data set. The bars are in descending order of absolute importance of the impact of features on the *y*-axis axis on the predicted value. A red bar indicates that the feature has a positive contribution to the predicted value. Conversely, a bar of a different color indicates that the feature has a negative contribution to the predicted value. The label on a bar indicates the deviation from the baseline model prediction value assigned to that parameter. For instance, in the case of the H2O model, the “Periodontal pockets = 1” feature made a marginal negative contribution of −0.17 to the deviation of the prediction from the base value of 0.635.

Both models interpret a high EQ-5D-5L score and low values of CPI and Periodontal pockets as low risks of metabolic syndrome, while high values of DMFT and CPI are interpreted as increased risks of metabolic syndrome.

**Figure 12:**
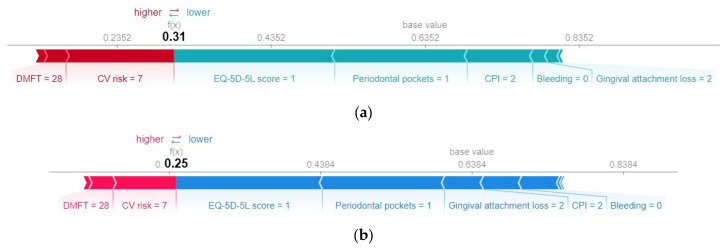
SHAP force plot for a healthy subject, without metabolic syndrome. (**a**) H2O AutoML (XGBoost), predicted probability = 0.31; (**b**) Auto-sklearn (RF), predicted probability = 0.25.

Further information regarding the actual values of the predictors for the specific case under analysis can be found in Figure 12, which displays the SHAP force graph. This graph is designed to offer insight into the relative significance of each feature’s contribution to the deviations in the prediction results. On the *x*-axis of the force plot, both the base value and the predicted value (*f(x)*) for the associated instance are indicated. The red bars situated on the left side of the value representing the model output correspond to the features that have made a positive contribution to the prediction’s deviation from the base value. Conversely, all bars located to the right of the value representing the model output denote features that contributed negatively to the prediction’s deviation from the base value.

Both models interpret the absence of bleeding and a relatively low value of gingival attachment loss as low risks of metabolic syndrome.

It can also be noted that for local interpretability, the importance of the predictors for particular cases may differ from the global importance of the feature variables (represented in Figure 6).

**Figure 13:**
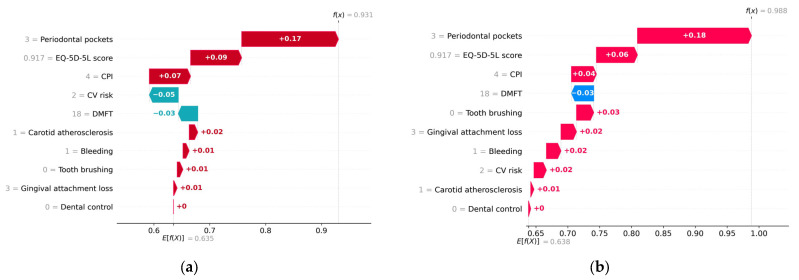
Waterfall plot for a patient diagnosed with metabolic syndrome. (**a**) H2O AutoML (XGBoost), predicted probability = 0.93; (**b**) Auto-sklearn (RF), predicted probability = 0.99.

**Figure 14:**
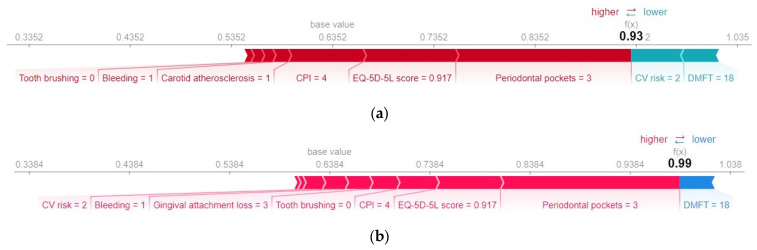
SHAP force plot for a patient diagnosed with metabolic syndrome. (**a**) H2O AutoML (XGBoost), predicted probability = 0.93; (**b**) Auto-sklearn (RF), predicted probability = 0.99.

Figure 13 and Figure 14 provide an example of a subject diagnosed with metabolic syndrome, estimated to have a probability of 0.93 of metabolic syndrome according to the H2O model and a probability of 0.99 according to the Auto-sklearn model.

Table 8 shows the average values of the prediction variables. Analyzing the prediction made for the patient with metabolic syndrome, it is found that lack of dental hygiene, the presence of bleeding, and carotid atherosclerosis have made a positive contribution to the predicted value (*True*). Above average values of the variables CPI, Periodontal pockets, and Gingival attachment loss increase the risk of metabolic syndrome. Both models interpret a relatively low (below average) value of EQ-5D-5L score with an increased risk of metabolic syndrome. In addition, a below-average DMFT value contributed negatively to the prediction (it is interpreted as low risk of metabolic syndrome).

It is worth noting the different treatment of the feature variable CV risk in the two ML models, in the analyzed case. The Auto-sklearn model considers that the CV risk predictor of value 2 has a positive contribution to the predicted value (*True*), while the H2O model considers that its contribution is negative.

However, this is not the cause for the four cases of erroneous classification (*FP*) by the Auto-sklearn (RF) model. For the respective cases, f(*x*) = 0.58 and CV risk = 9 still has a positive contribution to the predicted value, just like in the H2O model.

**Figure 15:**
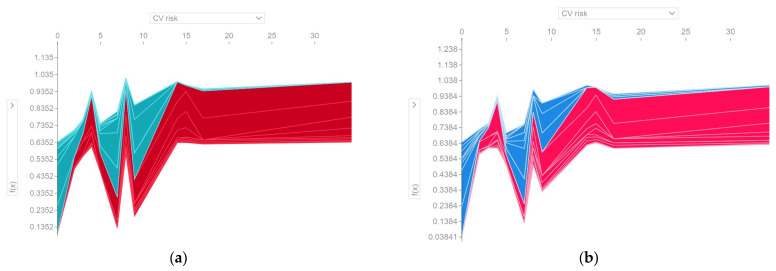
Contribution of the CV risk feature variable in the predictions made. *f(x)* represents the probability of predicting the *True* class (the existence of metabolic syndrome) for instance *x.* The red color indicates a positive contribution to the prediction of metabolic syndrome. (**a**) H2O AutoML (XGBoost); (**b**) Auto-sklearn (RF).

From Figure 15, it can be seen that both ML models exhibit the same pattern of the contribution of the CV risk feature variable in the predictions made. A CV risk value above 14 contributes positively to the deviation of the prediction from the base value and can be interpreted as an increased risk of metabolic syndrome. For a value lower than 14, the CV risk feature variable may have a positive or negative contribution to the prediction’s deviation from the base value, depending on the collective impact of the other predictors.

## 5. Discussion

Contemporary society, characterized by consumerism, has brought about profound changes in the lifestyles of a significant portion of the population, often without affording the human body adequate time to adapt to these new conditions. These transformations have led to a global surge in both cardiovascular and metabolic diseases, often with a frequent co-occurrence [73].

Unfortunately, the precise factors responsible for triggering and maintaining at the neuro-endocrine level of specific patterns of physiopathological and behavioral responses, which in turn lead to significant cardiovascular and metabolic changes from an early age, remain somewhat elusive [4,74].

Many authors emphasize the significant role of stress in triggering the onset of metabolic syndrome. This could disrupt the intricate neural mechanisms responsible for maintaining the delicate equilibrium between the sympathetic and parasympathetic nervous systems. These disruptions, along the hypothalamus-pituitary-adrenal gland pathway, may lead to the release of glucocorticoid hormones. The hormonal changes can trigger a cascade of events that lead to tissue resistance to insulin, ultimately contributing to the development of metabolic syndrome, cardiovascular diseases, proinflammatory alterations, and prothrombotic changes, resulting in increased morbidity [75,76].

A recent study [5], carried out on large samples of people, incriminates, in addition to the stress factor and reduced physical activity, the modern diet and a sedentary lifestyle in the occurrence of cardiometabolic diseases.

The application of artificial intelligence in healthcare, specifically through machine learning methods, has the potential to reveal hidden patterns and relationships by using highly optimized prediction models. In this study, six machine learning models were evaluated using medical data collected from 296 patients. The objective of the ML models was to differentiate with high accuracy between patients who were diagnosed with metabolic syndrome and those who were not, and to identify key predictors of metabolic syndrome.

To the best of our knowledge, this is the first study that employs AutoML frameworks for fine-tuning machine learning models to identify metabolic syndrome in subjects with periodontal disease. The main contribution of this paper, however, is to explain the outputs of machine learning models and highlight the most important predictors of metabolic syndrome using the SHAP analysis.

The findings show that H2O XGBoost outperforms other ML models, with a classification accuracy of 100%. Furthermore, we found that Periodontal pockets depth, CV risk, EQ-5D-5L score, CPI, and DMFT are the top five key predictors of metabolic syndrome. Other feature variables (Gingival bleeding, Daily tooth brushing, Gingival attachment loss) have notable, but lower, importance in the analyzed predictive models.

Concerning the frequent link between periodontal disease and metabolic syndrome, our findings, which indicate that the most robust associations are observed in relation to the severity of periodontal disease quantified by the depth of periodontal pockets, are consistent with results from other research studies [6,7]. Thus, the results obtained in the present study reject the null hypothesis (H_0_) stating that a higher periodontitis stage does not increase the incidence of metabolic syndrome. Our findings show that stages III and IV of periodontitis (periodontal pockets depth ≥ 6 mm) induce an increased incidence of metabolic syndrome, while for stage I (periodontal pockets depth ≤ 4 mm) a low incidence of metabolic syndrome is found.

The significant connections identified between health status, periodontal diseases, and the occurrence of metabolic syndrome, emphasized also in our research, may play a crucial role in disrupting the body’s homeostasis [77]. Metabolic syndrome increases the risk of type 2 diabetes, cardiovascular disease, and stroke. The presence of both cardiovascular disease and periodontal disease in an individual are associated with a lower quality of life [78]. From the perspective of personalized medicine, the assessment of these numerous interactions is vital in the development of tailored preventive and therapeutic strategies for individuals.

There are some limitations of our study. Firstly, because this is an observational retrospective study, it could not determine the exact order of onset of medical problems. Therefore, additional prospective studies are necessary to investigate the causal relationship between periodontal disease and metabolic syndrome. Secondly, the research was conducted at a single facility, namely Sibiu County Emergency Clinical Hospital. In future studies, it is advisable to carry out extensive external validation by utilizing data from multiple healthcare centers.

Another limitation of this study is the relatively small sample size. Consequently, further research with more substantial sample sizes is necessary to externally validate the applicability of our machine learning models.

Machine learning models are trained using historical data, and when applied in real-world scenarios, they can become outdated and experience a decline in accuracy over time. This phenomenon is known as drift and is caused by changes in the environment in which the model operates. To address this problem, as a future direction of development, we propose to create a framework that fully exploits the method of building ML models proposed in this study. An AutoML interface streamlines and automates the machine learning workflow and includes as pipeline stages automatic training and tuning of a wide selection of candidate models. The result of the AutoML run is a ranked list of optimized models that can be saved for later use in a production environment. Thus, the process of updating ML models can be automated. Regarding the interpretability of prediction models, the most appropriate option would be a universal SHAP explainer for any ML algorithm. The drawback of the KernelExplainer, used in this paper to compute the SHAP values, is its long running time. To treat this shortcoming, in our solution, the construction of fine-tuned machine learning models and the computation of SHAP values will be encapsulated within a task scheduled for execution at the designated time intervals or executed on demand. Through the data visualization component, the framework will provide support for the analysis of the predictions made and gaining insights into the prediction mechanisms of the machine learning models.

SHAP typically targets a technical audience (e.g., data scientists) for its explanations. However, translating graphical analyses into easily understandable terms poses a challenge, particularly when conveying information to non-technical individuals like medical experts or decision-makers. We intend to explore the potential integration of linguistic summarization into the proposed framework. This approach, as outlined in [79], facilitates the translation of model output explanations generated by SHAP into statements expressed in natural language. The goal is to enhance compatibility with the language used by medical experts, thereby making the explanations more accessible to this audience.

Furthermore, future prospective studies conducted over extended timeframes are essential to quantify the broader implications of preserving dental and periodontal health on overall body health.

## 6. Conclusions

In order to predict risk of metabolic syndrome in people with periodontal disease, in this study we created and evaluated six machine learning models. Our conclusion is that the H2O XGBoost model was the best performer, making correct predictions in all cases. Machine-learning-based prediction models can support healthcare professionals in clinical decision-making by helping them assess whether patients are at risk for metabolic syndrome. By using SHAP values and appropriate visualizations, information about how features in the data set influence the model output can be effectively analyzed, providing valuable insights and aiding model interpretation and decision making.

Our findings indicate an association between chronic periodontitis and metabolic syndrome, and they further suggest that the severity of chronic periodontitis has a high positive contribution to the occurrence of metabolic syndrome. ML models interpret a poor health status associated with a low EQ-5D-5L score as increased risks of metabolic syndrome.

Patients often underestimate the significance of maintaining good oral hygiene, scheduling regular dental check-ups, and taking preventive measures to manage periodontal disease. Consequently, there is a pressing need to reevaluate the interdisciplinary collaboration between specialized medical practitioners caring for individuals within high-risk groups and the attending dentists.

## Figures and Tables

**Table 1:** Previous studies on predictions involving metabolic syndrome and/or periodontal disease.

Paper	Data Set	Classifiers *	Metabolic Syndrome	Periodontal Disease	Explainability of the Prediction
[45]	18,553 patients from the Temple University Kornberg School of Dentistry predoctoral clinics	XGBoost	-	target	yes
[46]	1333 Taiwanese adult patients	DT	target	-	no
[47]	Metabolic data set from Kaggle repository, 12,012 records	SVM, KNN, DT, RF, AdaBoost, GB, SGB, CatBoost, XGBoost	target	-	yes
[48]	67,730 patients, Nanfang Hospital, China	XGBoost	target	-	yes
[49]	Tlalpan 2020 cohort study data set, Mexico City, 2289 subjects	RF, C4.5, DNN	target	-	no
[50]	Internal validation cohort, 6793 participants External validation cohort, 7681 participants	ANN, CART, SVM	target	-	no
[51]	KoGES cohort study, 3064 participants, Korea	KNN, Naïve Bayes, RF, DT, MLP, SVM	target	-	no
[52]	532 subjects, Toulouse University Hospital Centre, France	MLP	-	target	yes
[53]	Internal validation, 3453 participants, Taiwan External validation, 3685 participants, United States	AdaBoost, ANN, DT, GP, KNN, SVC, LDA, RF, Naïve Bayes	-	target	no
[54]	173,209 adults aged 40 years or older, South Korea	LR, DT, RF, XGBoost, TN	target	-	yes
[55]	2258 individuals	GBM, XGBoost, RF	feature	target	yes
[56]	6421 Japanese individuals	MLR	target	feature	no
[57]	DOME study, 132,529 subjects	LR, XGBoost	feature	feature	no
[58]	2401 samples From the NHANES database	LR, MLP, KNN, SVM, RF, XGBoost, Naïve Bayes	target	-	no
[59]	103 patients, Department of Internal Medicine, University of Catania, Italy	LR	target	feature	no
[60]	1011 participants, Brazil	LR	target	feature	no

* XGBoost = eXtreme Gradient Boosting, DT = Decision Tree, SVM = Support Vector Machine, KNN = K-Nearest Neighbors, RF = Random Forest, AdaBoost = Adaptive Boosting, GB = Gradient Boosting, SGB = Stochastic Gradient Boosting, CatBoost = Categorical Boosting, DNN = Deep Neural Network, ANN = Artificial Neural Networks, CART = Classification and Regression Tree, MLP = Multilayer Perceptron, GP = Gaussian Process, SVC = Support Vector Classification, LDA = Linear Discriminant Analysis, LR = Logistic Regression, TN = TabNet, GBM = Gradient Boosting Modeling, MLR = Multivariate Logistic Regression.

**Table 2:** Implementing a SHAP wrapper for Auto-sklearn models.

SHAP Wrapper for Auto-Sklearn Models	
Class	class SKLProbWrapper: def __init__(self, skl_model, feature_names): self.skl_model = skl_model self.feature_names = feature_names def predict_binary_prob(self, X): if isinstance(X, pd.Series): X = X.values.reshape(1,−1) self.dataframe= pd.DataFrame(X, columns=self.feature_names) self.predictions = self.skl_model.predict_proba(self.dataframe.values) return self.predictions.astype(‘float64’)[:,−1] #probability of True class
Use case	skl_wrapper = SKLProbWrapper(model, dframe.columns) skl_explainer = shap.KernelExplainer(skl_wrapper.predict_binary_prob, dframe) shap_values = skl_explainer(dframe)

**Table 3:** The assignment of numerical values to categorical variables.

Feature Variable	Recorded Values	Assigned Numerical Values	Count (Total = 296)	Percentage %
Gingival bleeding	no	0	104	35.14
	yes	1	192	64.86
Periodontal pockets depth	-	0	96	32.43
	≤3.5	1	68	22.97
	>3.5	2	96	32.43
	>5	3	36	12.17
Carotid atherosclerosis	no	0	158	53.38
	yes	1	138	46.62
Dental control	no	0	248	83.78
	yes	1	48	16.22
Daily tooth brushing	irregular/occasional	0	84	28.38
	1 per day	1	136	45.94
	2 per day	2	76	25.68

**Table 4:** Definition of the confusion matrix elements.

TN	(True Negative): the value of correct predictions of negatives out of actual negative cases.
TP	(True Positive): the value of correct predictions of positives out of actual positive cases.
FP	(False Positive): the value of incorrect positive predictions.
FN	(False Negative): the value of incorrect negative predictions.

**Table 5:** Performance measures in machine learning classification models.

$Accuracy=\frac{TN+TP}{TN+FP+TP+FN}$	$Precision=\frac{TP}{TP+FP}$
$Recall=\frac{TP}{TP+FN}$	$F1\;Score=2*\frac{Precision*Recall}{Precision+Recall}$
$Specificity=\frac{TN}{TN+FP}$	$Balanced\;accuracy=\frac{Recall+Specificity}{2}$

**Table 6:** The best models built with AutoML frameworks.

AutoML Framework	Algorithm	Model Parameters and Hyperparameters
H2O AutoML	XGBoost	number_of_trees: 47, max_depth: 10, min_rows: 5, min_child_weight: 5, learn_rate: 0.3, eta: 0.3, sample_rate: 0.6, normalize_type: tree, distribution: bernoulli, grow_policy: depthwise, dmatrix_type: dense, booster: gbtree
H2O AutoML	DRF	number_of_trees: 32, number_of_internal_trees: 32, model_size_in_bytes: 5639, min_depth: 4, max_depth: 7, mean_depth: 5.75, min_leaves: 7, max_leaves: 15, mean_leaves: 9.375
H2O AutoML	GBM	number_of_trees: 628, number_of_internal_trees: 628, model_size_in_bytes: 115250, min_depth: 1, max_depth: 7, mean_depth: 4.915605, min_leaves: 2, max_leaves: 15, mean_leaves: 9.91242
Auto-sklearn	RF	bootstrap: True, criterion: ‘gini’, max_depth: ‘None’, max_features: 0.5, max_leaf_nodes: ‘None’, min_impurity_decrease: 0, min_samples_leaf: 1, min_samples_split: 2, min_weight_fraction_leaf: 0
Auto-sklearn	MLP	activation_function: relu, alpha: 0.02847755502162456, beta_1: 0.9, beta_2: 0.999, early_stopping: train, epsilon: 10^−8^, hidden_layer_depth: 2, learning_rate_init: 0.000421568792103947, num_nodes_per_layer: 123, shuffle: True, solver: adam
Auto-sklearn	ExtraTrees	bootstrap: False, criterion: entropy, max_features: 0.993803313878608, max_leaf_nodes: None, min_impurity_decrease: 0, min_samples_leaf: 2, min_samples_split: 20, min_weight_fraction_leaf: 0,

**Table 7:** The values of the performance metrics of the prediction models.

AutoML Framework	Model	Precision	Recall	Accuracy	Specificity	Balanced Accuracy	F1	Incorrect Classifications
H2O	XGBoost	1	1	1	1	1	1	0
	DRF	1	0.885	0.932	1	0.942	0.939	*FN* = 6
	GBM	1	0.769	0.864	1	0.885	0.870	*FN* = 12
Auto-sklearn	RF	0.929	1	0.955	0.889	0.944	0.963	*FP* = 4
	MLP	0.897	1	0.932	0.833	0.917	0.945	*FP* = 6
	ExtraTrees	0.867	1	0.909	0.778	0.889	0.929	*FP* = 8

**Table 8:** The mean values of the feature variables.

Feature	Mean Value
DMFT	21.954
CPI	2.682
Periodontal pockets	1.5
Bleeding	0.636 *
Tooth brushing	0.818
Dental control	0.136 *
Gingival attachment loss	2.454
CV risk	7.636
Carotid atherosclerosis	0.477 *
EQ-5D-5L score	0.935

* Binary variable.

## Data Availability

The data presented in this study are available on request from the corresponding author. The data are not publicly available due to confidentiality restrictions. The implementation details for obtaining the results presented in study can be found at https://github.com/automl-mets/MetS (accessed on 26 November 2023).
